# Burden of malaria in the Comoros, 1990–2021: findings from the global burden of disease study 2021

**DOI:** 10.3389/fpubh.2025.1470021

**Published:** 2025-03-12

**Authors:** Sheng Zhou, Jiarui Zhang, Chengcheng Li

**Affiliations:** ^1^Artemisinin Research Center, Guangzhou University of Chinese Medicine, Guangzhou, China; ^2^School of Public Health and Management, Guangzhou University of Chinese Medicine, Guangzhou, China; ^3^Department of Health Service Management, Humanities and Management School, Zhejiang Chinese Medical University, Hangzhou, China

**Keywords:** malaria, disease burden, decomposition analysis, trend analysis, GBD 2021

## Abstract

**Background:**

Malaria is a major public health issue in the Comoros. Analyzing the malaria burden will aid in designing prevention, control, and eradication strategies based on scientific evidence.

**Methods:**

We retrieved data from the 2021 Global Burden of Disease (GBD) database to assess the malaria burden in the Comoros in 2021, stratified by sex and age. The indicators used to measure this burden included the absolute numbers and rates of incidence, prevalence, and mortality, as well as disability-adjusted life years (DALYs), years of life lost (YLLs), and years lived with disability (YLDs). Decomposition analysis was used to quantify the contributions of demographic aging, population growth, and epidemiological changes to the malaria burden from 1990 to 2021. Joinpoint regression analysis was employed to examine temporal trends in the malaria burden over this period.

**Results:**

In 2021, females, particularly those under 40, had a higher overall malaria burden than males, except for the age-standardized incidence rate (ASIR) and the age-standardized mortality rate (ASMR). Individuals under 30 years of age experienced approximately 52% of new malaria episodes, 68% of prevalent cases, and 62% of YLDs. Children under 5 and those aged 15 to 30 accounted for about 41% of malaria-related deaths, 54% of YLLs, and 53% of DALYs. Between 1990 and 2021, the malaria burden in the Comoros declined substantially, with age-standardized incidence (ASIR), prevalence (ASPR), mortality (ASMR), and DALY rates (ASDR) decreasing by over 85%. Decomposition analysis indicated that epidemiological changes played a pivotal role in reducing disease burden. Over the past 32 years, the average annual percentage change (AAPC) in the ASPR was statistically significant at −7.60% (t = −2.68, *p* < 0.05). Moreover, the annual percentage change (APC) in ASIR and ASPR showed the most significant decline from 2012 to 2015, with APCs of −70.47% (t = −3.01, *p* < 0.05) and − 66.55% (t = −14.94, *p* < 0.05), respectively.

**Conclusion:**

This study indicates that women under 40, school-aged children, and adults under 30 in the Comoros bear a higher malaria burden. Although current malaria control measures are effective, achieving a malaria-free status will require integrated strategies.

## Introduction

Malaria is an infectious disease caused by *Plasmodium* parasites and transmitted by female *Anopheles* mosquitoes. Of the various *Plasmodium* species infecting humans, *Plasmodium falciparum* is the most lethal and the leading cause of malaria-related deaths worldwide (1). Although malaria is both preventable and treatable, it remains a critical public health concern in endemic regions, where it imposes a heavy toll on human health and socioeconomic development. Globally, the incidence of malaria remains far from optimistic. In 2022, an estimated 249 million malaria cases and 0.61 million malaria-related deaths were reported, thereby posing severe threats to global health (2). Severe malaria can lead to life-threatening complications such as severe anemia, acute kidney failure, cerebral malaria, pulmonary edema, and hemorrhage, all of which are closely associated with elevated mortality rates (3). For instance, cerebral malaria alone accounts for approximately 20% of adult deaths and 15% of child deaths (4). Moreover, malaria also negatively affects the economic stability of countries where it is endemic (5). Studies have shown that populations living below the poverty line in sub-Saharan Africa face an elevated risk of *Plasmodium* infection (6, 7), thereby perpetuating a vicious cycle between poverty and malaria prevalence. Because malaria remains a significant barrier to both global health and economic progress, sustained and integrated efforts in prevention, control, and eradication are crucial, especially in highly affected regions.

The Comoros archipelago, a key malaria-endemic area, is located north of Madagascar, between 11°20′ and 13°04′ South latitude and between 42° and 45° East longitude. The archipelago comprises four islands: Mayotte, Mohéli, Anjouan, and Grande Comore, with the latter three forming the Union of the Comoros (8). There are two distinct seasons: a hot and rainy season from November to April and a dry, cooler season from May to October. Malaria transmission in the Comoros shows both perennial and seasonal patterns, peaking during the rainy season (9). The World Malaria Report 2023 indicates that malaria still has a substantial impact on the Comoros. In 2022, 20,675 malaria cases were reported, nearly twice the number of cases documented in 2021. Moreover, relative to 2015, the mortality rate in 2022 increased by at least 55% (2). Hence, the malaria burden in the Comoros remains significant. However, few comprehensive analyses of the malaria burden in the Comoros have been conducted. In this study, we used data from the GBD 2021 database to provide an in-depth examination of the malaria burden by sex and age in 2021. We also assessed the contributions of demographic and epidemiological factors to this burden and investigated trends from 1990 to 2021. These findings will offer key insights to policymakers in the Comoros and help guide more effective malaria prevention, control, and elimination strategies.

## Methods

### Overview

The 2021 Global Burden of Disease (GBD) database employs multiple modeling strategies, including DisMod-MR 2.1, the Cause of Death Ensemble Model (CODEm), and spatiotemporal Gaussian process regression (STGPR), to generate consistent estimates of incidence, prevalence, mortality, and DALYs for 371 diseases and injuries across 204 countries and territories (10, 11). All estimates are presented as absolute numbers, percentages (%), and rates (per 100,000 population). The 95% uncertainty interval (UI) is calculated by taking the 2.5th and 97.5th percentiles from an ordered set of 500 draws (10). This approach allows for robust estimates of the global disease burden while enabling valid comparisons across geographic regions, populations, and periods. A detailed description of the GBD 2021 methodology has been published elsewhere (10).

### Definitions

Malaria is defined as a life-threatening disease caused by *Plasmodium* parasites, which are transmitted to humans through the bites of infected female *Anopheles* mosquitoes (1). The species most frequently implicated in human infections are *Plasmodium falciparum*, *Plasmodium vivax* (*P. vivax*), *Plasmodium malariae* (*P. malariae*), *Plasmodium ovale* (*P. ovale*), and *Plasmodium knowlesi* (*P. knowlesi*) (12). According to the International Classification of Diseases, 11th Edition (ICD-11), malaria is classified under the codes 1F42, 1F45, 1F44, 1F40, 1F43, 1F41, and KA64.Y.^1^ According to the UK malaria treatment guidelines 2016, any individual presenting with a fever or a recent history of fever after returning from or visiting a malaria-endemic region is suspected of having malaria (13). A diagnosis is confirmed when blood test results are positive for malaria parasites.

### Data sources

We extracted malaria burden data for the Comoros from the Global Health Data Exchange (GHDx) query tool^2^ for the period 1990–2021, stratified by sex and age. The indicators included the number of new malaria episodes, prevalent cases, deaths, disability-adjusted life years (DALYs), years of life lost (YLLs), and years lived with disability (YLDs), as well as their corresponding crude rates, age-standardized rates, and 95% uncertainty intervals (UIs). Age groups were categorized into seventeen 5-year intervals (<5 years, 5 to 9 years, 10 to 14 years, 15 to 19 years, and up to 80+ years). YLDs = (number of malaria incident cases or episodes × average duration) × disability weights. YLDs represented the number of years spent in a sub-optimal state of health as a result of malaria infection. YLLs = number of deaths × life expectancy at the age of death. YLLs denoted the number of years of life lost due to premature mortality from malaria relative to the expected lifespan. DALYs were a key metric for quantifying the overall health loss resulting from both fatal and nonfatal conditions attributable to malaria infection. They were calculated by summing the YLLs with the YLDs (10, 14).

### Data analysis

Excel (version 6.8.2) and R software (version 4.3.2) were used to enter and organize the 2021 Comoros malaria data, stratified by sex and age. The decomposition method, described in detail elsewhere (15, 16), was applied to assess the individual contributions of demographic aging, population growth, and epidemiological changes to the malaria burden, measured as the number of new malaria episodes, prevalent cases, deaths, and DALYs, from 1990 to 2021. If the value of a decomposition component exceeded 0, it was interpreted as contributing to an increase in the malaria burden; if it was below 0, it was interpreted as contributing to a decrease. Joinpoint regression analysis (17) was conducted to detect significant changes in the temporal trends of the age-standardized incidence rate (ASIR), age-standardized prevalence rate (ASPR), age-standardized mortality rate (ASMR), and age-standardized DALY rate (ASDR) over the period of 1990 to 2021. This analysis employed the average annual percent change (AAPC) and annual percent change (APC) to quantify both overall and specific-period trends. The optimal number of joinpoints was determined through a grid search and permutation test. Statistical significance was defined as *p*-values below 0.05, whereby *p* < 0.05 indicated a statistically significant upward or downward trend, and *p* > 0.05 indicated no statistically significant trend. Integrating decomposition and joinpoint regression analyses allowed for a clearer understanding of the underlying drivers of malaria burden changes, as well as the detection of statistically significant temporal shifts.

The decomposition analysis was performed in R software (version 4.3.2) using the packages “dplyr,” “data.table,” “purrr,” “tidyr,” “ggsci” and “ggplot2.” The joinpoint analysis was conducted using joinpoint regression software (version 5.2.0, 2023).

## Results

### Sex and age-specific malaria burden in the Comoros: a 2021 descriptive analysis

**Figure 1:**
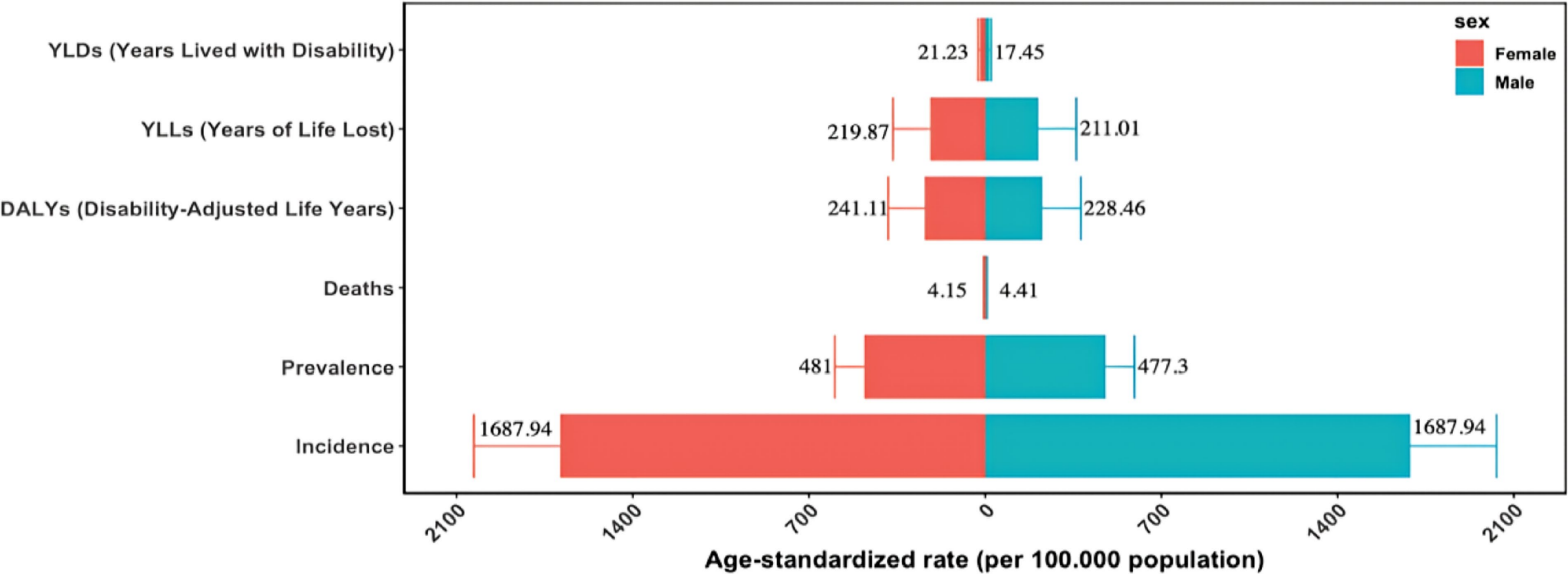
Gender disparities in the burden of malaria in the Comoros in the year 2021.

Figure 1 and Supplementary Table 1 present our findings on gender disparities in the malaria burden in the Comoros for 2021 as measured by the ASIR, ASPR, ASMR, ASDR, age-standardized YLL rate, and age-standardized YLD rate. Overall, in 2021, females bore a higher malaria burden than males, except for the ASIR and ASMR. Specifically, no difference was observed in the ASIR between males and females, with both sexes exhibiting an ASIR of 1,687.94 per 100,000 population (95% uncertainty interval [UI]: 1,395.05 to 2,031.47 per 100,000). Males exhibited a higher ASMR, at 4.41 per 100,000 (95% UI: 1.87 to 7.67), compared to 4.15 per 100,000 for females (95% UI: 1.95 to 6.94). Moreover, the ASPR, ASDR, age-standardized YLL rate, and age-standardized YLD rate in females were 1.01, 1.06, 1.04, and 1.22 times those observed in males, respectively. These values corresponded to 481 per 100,000 (95% UI: 35.04 to 597.47) for the ASPR, 241.11 per 100,000 (95% UI: 125.55 to 386.05) for the ASDR, 219.87 per 100,000 (95% UI: 104.11 to 366.33) for the age-standardized YLL rate, and 21.23 per 100,000 (95% UI: 13.96 to 28.38) for the age-standardized YLD rate. Notably, DALYs were predominantly attributable to YLLs (approximately 92% of total DALYs) rather than YLDs (approximately 8%).

**Figure 2:**
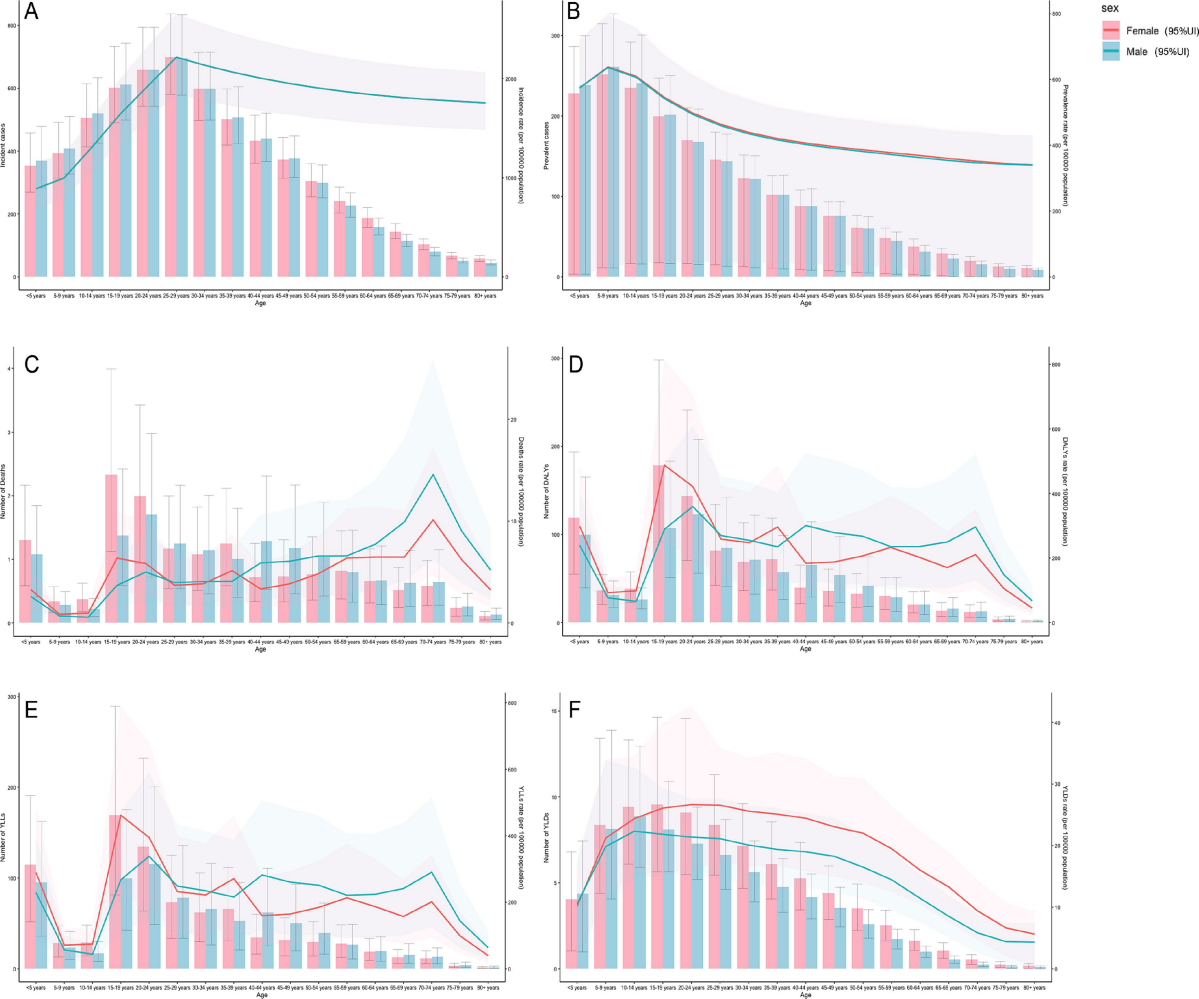
Gender disparities in the burden of malaria in different age groups. **(A)** Incidence. **(B)** Prevalence. **(C)** Mortality. **(D)** DALYs (disability-adjusted life years). **(E)** YLLs (years of life lost). **(F)** YLDs (years lived with disability). 95%Ul, 95% uncertainty interval.

Figure 2 presents the gender disparities and variations in the malaria burden across different age groups. According to the 2021 incidence data, a concerning increase in both the number of new malaria episodes and the crude incidence rate (CIR) was observed among individuals under 30. For both males and females, the peak values of new malaria episodes and CIR occurred in the 25 to 29 age group, followed by a decline after age 30. The lowest number of new malaria episodes was recorded in individuals aged over 80, whereas the lowest CIR was observed in children under 5. Before the age of 50, males generally had more new malaria cases than females; however, this trend reversed in age groups older than 50. Notably, no obvious gender discrepancy in the CIR was detected (Figure 2A; Supplementary Table 2). Based on 2021 prevalence data, children under 10, regardless of gender, experienced increases in both malaria cases and the crude prevalence rate (CPR), with the highest values observed in the 5 to 9 age group. From age 10 onward, both malaria cases and CPR steadily decreased for both males and females, reaching their lowest levels among individuals over 80. Before the age of 20, males had higher numbers of malaria cases, but after age 20, females showed relatively higher case numbers. Moreover, the overall CPR was slightly higher in females than in males (Figure 2B; Supplementary Table 2).

In 2021, the highest number of deaths among males occurred in the 20 to 24 age group, while for females, it occurred in the 15 to 19 age group. In contrast, both genders exhibited the lowest number of deaths in individuals aged over 80. Females experienced more deaths than males before age 25; however, this pattern reversed after age 25. The overall crude mortality rate (CMR) initially decreased, then fluctuated with an increasing trend, and ultimately declined. The 70 to 74 age group recorded the highest CMR for both genders. The lowest CMR for males was observed in the 10 to 14 age group, whereas for females, it was observed in the 5 to 9 age group. Below age 40, females had a higher overall CMR than males, but this disparity reversed in individuals older than 40 (Figure 2C; Supplementary Table 2). During the same period, for both genders, the numbers of DALYs and YLLs, as well as their corresponding crude rates, initially decreased, then increased, and ultimately declined overall, despite some fluctuations. The highest values for these metrics were observed among 20 to 24-year-old males and 15 to 19-year-old females, whereas individuals aged over 80 had the lowest values. Notably, as age increased, the crude rates for DALYs and YLLs in males remained relatively high, whereas those in females stayed comparatively lower (Figures 2D,E; Supplementary Table 2). Regarding YLDs, both the number of YLDs and the crude YLD rate for males and females initially rose and then steadily declined with advancing age. Males aged 10 to 14 recorded the highest YLD values and crude YLD rate, whereas females reached their peak YLD values in the 15 to 19 age group and recorded the highest crude YLD rate in the 20 to 24 age group. The lowest YLD values and crude YLD rate were found in individuals aged over 80. Overall, females exhibited higher YLD values and crude YLD rates than males (Figure 2F; Supplementary Table 2).

### Changes in the burden of malaria between 1990 and 2021

Over the past 32 years, the burden of malaria in the Comoros, as measured by the ASIR, ASPR, ASMR, and ASDR, showed an overall declining trend, with all indicators experiencing reductions exceeding 85% between 1990 and 2021. In 1990, the ASIR was 18,092.65 per 100,000 population (95% UI: 379.87 to 89,671.92). By 2021, it decreased by 90.67%, reaching 1,687.94 per 100,000 (95% UI: 1,395.05 to 2,031.47). Similarly, the ASPR declined from 6,634.64 per 100,000 (95% UI: 135.23 to 48,207.39) in 1990 to 479.17 per 100,000 (95% UI: 33.14 to 595.59) in 2021, representing a 92.78% reduction over the study period. Meanwhile, the ASMR dropped by 89.97%, from 42.47 per 100,000 (95% UI: 0.56 to 219.77) in 1990 to 4.26 per 100,000 (95% UI: 1.90 to 7.24) in 2021. In addition, the ASDR decreased from 2,417.39 per 100,000 (95% UI: 75.01 to 11,517.29) in 1990 to 234.27 per 100,000 (95% UI: 116.63 to 380.55) in 2021, marking a 90.31% reduction from the 1990 level. Over the entire study period, the most pronounced decline in the malaria burden occurred between 2010 and 2021, followed by the period between 1990 and 1999 (Table 1).

**Table 1:** Changes in the burden of malaria in the Comoros, 1990–2021.

Measure	1990	1999	1990–1999	2000	2009	2000–2009	2010	2021	2010–2021	1990–2021
	Rate per 10^5^ (95% UI)	Rate per 10^5^ (95% UI)	RCR (%)	Rate per 10^5^ (95% UI)	Rate per 10^5^ (95% UI)	RCR (%)	Rate per 10^5^ (95% UI)	Rate per 10^5^ (95% UI)	RCR (%)	RCR (%)
ASIR	18092.65 (379.87 to 89671.92)	13456.3 (237.72 to 81524.28)	−25.63	11993.94 (332.4 to 67648.12)	8725.81 (7178.74 to 10639.6)	−27.25	17200.31 (14327.51 to 20935.66)	1687.94 (1395.05 to 2031.47)	−90.19	−90.67
ASPR	6634.64 (135.23 to 48207.39)	4937.75 (320.89 to 31475.57)	−25.58	5261.45 (118.29 to 44006.63)	5314.65 (4441.89 to 6424.3)	1.01	6238.1 (4942.81 to 8020.06)	479.17 (33.14 to 595.59)	−92.32	−92.78
ASMR	42.47 (0.56 to 219.77)	33 (0.54 to 195.21)	−22.30	31.75 (1.21 to 129.41)	63.27 (31.82 to 110.12)	99.28	86.34 (44.15 to 139.7)	4.26 (1.9 to 7.24)	−95.07	−89.97
ASDR	2417.39 (75.01 to 11517.29)	1853.73 (88.22 to 10113.29)	−23.32	2027.71 (123.85 to 7612.05)	3368.65 (1792.7 to 5696.94)	66.13	5011.98 (2,743 to 7741.25)	234.27 (116.63 to 380.55)	−95.33	−90.31

ASIR, age-standardized incidence rate. ASPR, age-standardized prevalence rate. ASMR, age-standardized mortality rate. ASDR, age-standardized DALY rate. 95% UI: Uncertainty Intervals. RCR: relative change rate (%) = (observed value- initial value)/initial value × 100%. DALYs, disability-adjusted life years.

### Decomposition analysis of the malaria burden

**Figure 3:**
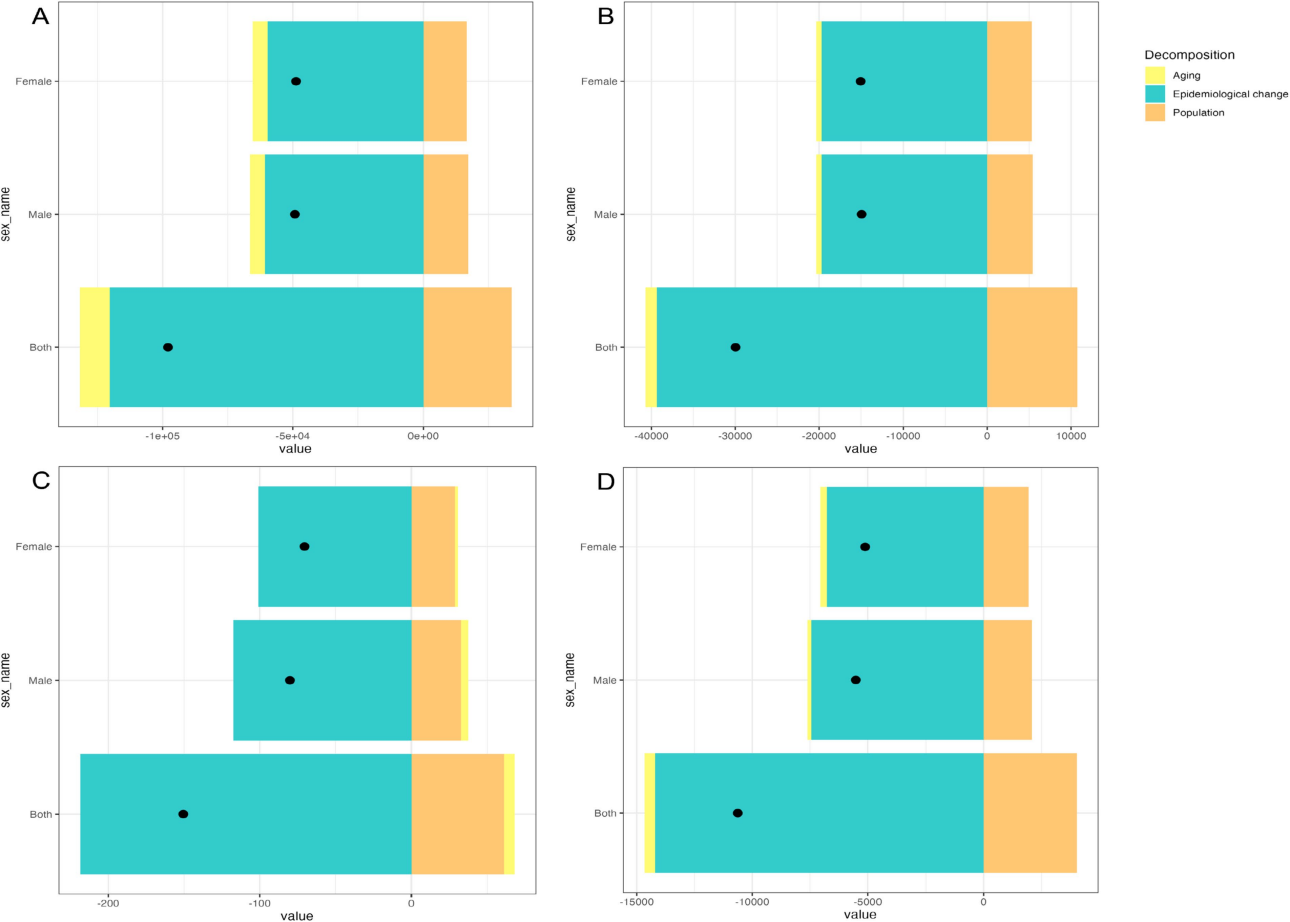
Decomposition analysis of the malaria burden in the Comoros (1990 to 2021): **(A)** Incidence. **(B)** Prevalence. **(C)** Deaths. **(D)** DALYs (disability-adjusted life years). The black dot indicates the overall change attributable to aging, population growth, and epidemiological shifts.

Figure 3 illustrates the decomposition analysis of the malaria burden in the Comoros from 1990 to 2021. During the study period, all malaria burden indicators, including the number of new malaria episodes, prevalent cases, deaths, and DALYs, decreased significantly. In 2021, new malaria cases decreased to 12,384 (95% UI: 10,210 to 14,942), representing an 88.78% reduction compared to 1990. Similarly, the number of prevalent cases declined to 3,674 (95% UI: 258 to 4,565), showing an 89.08% decrease. Malaria-related deaths decreased to 30 (95% UI: 13 to 50), marking an 83.33% reduction, while DALYs fell to 1,778 (95% UI: 886 to 2,878), representing an 85.67% decline (Supplementary Table 3). These substantial reductions in the malaria burden across both sexes were predominantly attributable to epidemiological changes. Epidemiological shifts contributed 122.78, 131.36, 145.19, and 133.67% to the reductions in new malaria episodes, prevalent cases, deaths, and DALYs, respectively, between 1990 and 2021. Conversely, population growth contributed 34.57, 35.86, 40.64, and 37.94% to the increases in these same indicators. Demographic aging demonstrated variable effects on the malaria burden. Specifically, it contributed 11.79, 4.50, and 4.27% to the reductions in the number of new malaria episodes, prevalent cases, and DALYs, respectively, while simultaneously contributing 4.55% to the increase in malaria-related deaths (Figure 3; Supplementary Table 4).

### Joinpoint regression analysis of the burden of malaria between 1990 and 2021

Table 2 illustrates the trends in malaria burden in the Comoros from 1990 to 2021, as measured by ASIR, ASPR, ASMR, and ASDR. The overall change in ASIR from 1990 to 2021 was not statistically significant. The ASIR trend was divided into four distinct periods: a significant increase between 1990 and 2012 (APC = +13.63%, t = 3.21, *p* < 0.05), followed by a dramatic decrease from 2012 to 2015 (APC = −70.47%, t = −3.01, *p* < 0.05), then a resurgence between 2015 and 2019 (APC = +85.29%, t = 2.90, *p* < 0.05), and finally a non-significant change from 2019 to 2021 (APC = −48.18%, t = −1.41, *p* = 0.17) (Supplementary Table 5). The ASPR demonstrated an overall downward trend with periodic fluctuations (AAPC = −7.60%, t = −2.68, *p* < 0.05), declining from 6,634.64 per 100,000 in 1990 to 479.17 per 100,000 in 2021, representing a 92.78% reduction. This trend was characterized by several distinct periods. Specifically, during the early period from 1990 to 2005, a downward trend was observed (APC = −8.09%, t = −2.35, *p* < 0.05) with some fluctuations, reducing malaria cases by 81.41% from the 1990 figures. Subsequently, a pronounced increase between 2005 and 2009 was observed (APC = +42.01%, t = 9.71, *p* < 0.05), with malaria cases spiking by 330.79% from the 2005 levels. Although there was a slight increase from 2009 to 2012, it did not reach statistical significance (APC = 1.81%, t = 0.16, *p* = 0.87). The most remarkable decline was observed between 2012 and 2015 (APC = −66.55%, t = −14.94, *p* < 0.05), representing a 95.66% reduction as values decreased from 4,368.45 per 100,000 in 2012 to 189.73 per 100,000 in 2015. Subsequently, a rebound occurred between 2015 and 2018, during which the largest magnitude of increase in malaria cases was observed (APC = 74.13%, t = 4.12, *p* < 0.05). Malaria cases increased from 189.73 per 100,000 in 2015 to 1,230.11 per 100,000 in 2018, representing a 548.35% increase over 2015 levels. Nonetheless, during the most recent period from 2018 to 2021, a further decrease was observed (APC = −28.86%, t = −2.24, *p* < 0.05), reflecting a 61.05% reduction from the 2018 levels (Supplementary Table 5). The overall trends in both ASMR and ASDR from 1990 to 2021 were not statistically significant. While both indicators showed statistically significant increases between 1990 and 2012, changes in subsequent periods did not reach statistical significance (Supplementary Table 5).

**Table 2:** Joinpoint regression analysis of the burden of malaria in the Comoros, 1990-2021.

ASIR		ASPR			ASMR		ASDR	
Period	APC (%) 95% CI	Period	APC (%) 95% CI	AAPC (%) 95% CI	Period	APC (%) 95% CI	Period	APC (%) 95% CI
1990-2012	13.63 (4.60 to 23.44)	1990-2005	–8.09 (–14.84 to –0.80)	–7.60 (-12.80 to -2.10)	1990-2012	7.68 (1.19 to 14.58)	1990–2012	7.98 (1.55 to 14.81)
2012-2015	–70.47 (–87.27 to –31.50)	2005-2009	42.01 (31.49 to 53.38)					
2015-2019	85.29 (19.02 to 188.46)	2012-2015	–66.55 (–71.39 to –60.89)					
		2015-2018	74.13 (30.73 to 131.94)					
		2018-2021	–28.86 (–48.51 to –1.70)					

ASIR, age-standardized incidence rate; ASPR, age-standardized prevalence rate; ASMR, age-standardized Mortality rate; ASDR, age-standardized DALYs rate; APC, annual percent change; AAPC, average annual percent change presented for full period; 95% CI: 95% confidence interval.

## Discussion

The Global Technical Strategy for Malaria 2016 to 2030 (GTS), adopted in 2015 and revised in 2021, sets priorities for reducing malaria morbidity and mortality while gradually eliminating malaria in countries with persistent transmission. Although the strategy established an ambitious target of reducing global malaria mortality and incidence by 90% by 2030 (18), recent epidemiological data suggest major challenges in achieving these objectives. In 2022, there were approximately 249 million malaria cases and 610,000 malaria-related deaths worldwide, with 95.4% of fatalities recorded in the WHO African Region (2). The situation in the Comoros is particularly concerning, as reported infections almost doubled from 10,537 cases in 2021 to 20,675 in 2022, contributing significantly to the global resurgence (2). In this study, we have comprehensively analyzed the malaria burden in the Comoros from 1990 to 2021.

Analysis of the malaria burden in the Comoros in 2021 revealed important demographic patterns. Females had a higher malaria burden than males (Figure 1), partly because pregnant women are biologically more susceptible to *Plasmodium falciparum* infection and tend to experience severe disease (19, 20). This group faces a dual challenge: infected pregnant women are at increased risk of severe complications, including respiratory distress, cerebral malaria, and mortality (21), while simultaneously serving as reservoirs of malaria parasites in the community (19). Intermittent preventive treatment in pregnancy (IPTp) and the use of long-lasting insecticide-treated nets (LLINs) have been proven to effectively prevent malaria in pregnant women (22–24). Priority interventions, such as IPTp and LLIN distribution, are therefore essential for mitigating risks in this demographic. Expanded antenatal malaria screening and guaranteed access to free treatment for febrile illnesses for pregnant women are also recommended. Age-specific analyses identified additional high-risk groups. Approximately 32% of new malaria cases in 2021 occurred among individuals aged 15 to 30, with the 25 to 29 age group alone accounting for 11% of total incident cases (Supplementary Table 6). This pattern may be explained by two factors: increased outdoor occupational or recreational activity among young adults, which raises their exposure to malaria-infected mosquitoes; and the high proportion of women of reproductive age in this group. It is therefore recommended that individuals wear long-sleeved shirts and long pants when outdoors, while national health authorities should implement early screening for malaria in pregnant women. Meanwhile, children under five remained disproportionately affected, accounting for 6% of new cases in the Comoros (Supplementary Table 6). This finding is consistent with global trends in malaria prevalence among children (2). Given the demonstrated efficacy of vaccines in African children, such as the RTS, S/AS01 and R21/Matrix-M vaccines, which can reduce the risk of *P. falciparum* infection and malaria-related mortality (12), introducing such vaccines in the Comoros is worth consideration. School-aged children (5–14 years) emerged as another high-risk demographic, representing 27% of total prevalent cases in 2021 (Supplementary Table 7), which was consistent with previous research findings (25). Recent studies show that preventive therapies reduce *P. falciparum* prevalence, anemia incidence, and subsequent clinical malaria risk in this age group (25, 26). In the Comoros, school-aged children remain frequently excluded from targeted interventions. This situation necessitates several interventions: strengthening school-based preventive treatments and malaria education, expanding healthcare accessibility for school-aged children, and enhancing vector control measures through LLIN distribution and indoor residual spraying (IRS). The 15-30-year-old demographic accounted for 33% of malaria deaths (Supplementary Table 8), partly attributable to the higher proportion of new malaria episodes within this age range. The elevated mortality rate may also be explained by the higher proportion of pregnant women in this demographic. Kochar et al. identified that pregnant women constitute a high-risk group for malaria infection and experience higher mortality rates compared to men and other age groups (27). The distribution of DALYs, YLLs, and YLDs across age groups further substantiated the heightened malaria burden in this demographic (Supplementary Tables 9–11). These findings indicate that the malaria burden in the Comoros has expanded beyond children under five (28) to affect school-aged children and adults, particularly women under 40.

Decomposition analysis revealed that epidemiological changes have played a crucial role in reducing the malaria burden over the study period, underscoring the importance of effective prevention and treatment strategies. Although demographic aging contributed to lower overall incidence and prevalence rates through enhanced immune responses against *Plasmodium* parasites, it was associated with increased malaria-related mortality among older adults due to diminished immunity. Furthermore, aging resulted in reduced DALYs, as malaria cases typically declined with age. Conversely, population growth contributed to increases in all malaria burden indicators, primarily because children who lack immunity to *Plasmodium* remain the most susceptible group, facing elevated mortality risks (29).

**Figure 4:**
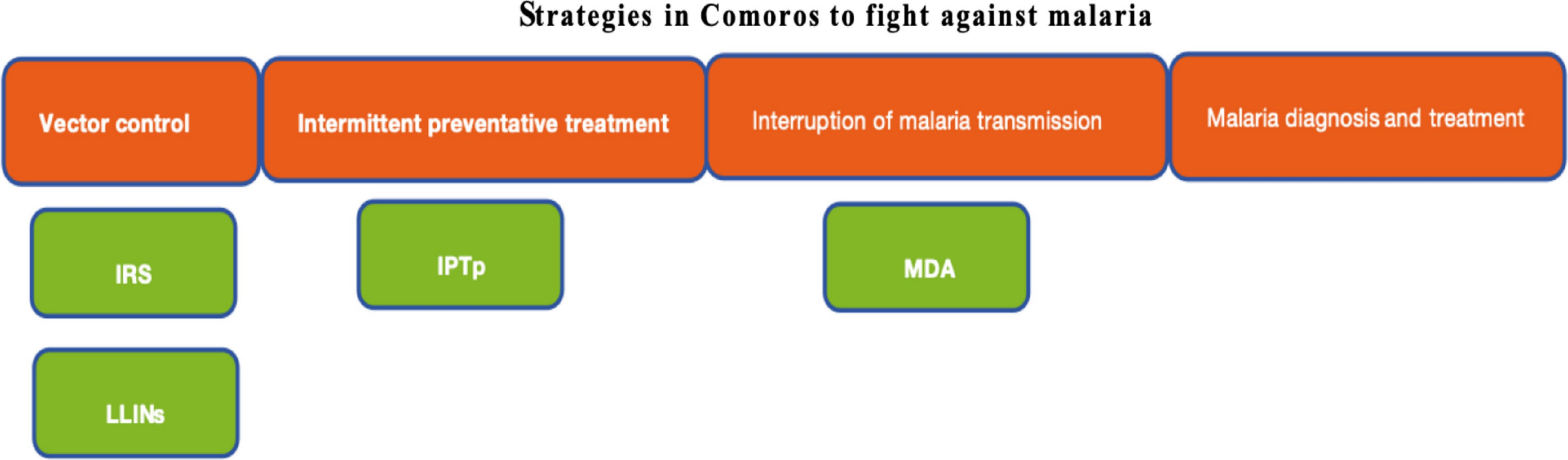
Strategies in the Comoros to fight against malaria. LLINs, longlasting insecticidal nets. IRS, Indoor residual spraying. IPTp, Intermittent preventive treatment of malaria in pregnancy. MDA, Mass drug administration.

Based on existing malaria prevention and control measures in the Comoros (Figure 4), significant progress has been made in reducing the malaria burden over the past 32 years, with all age-standardized indicators, including the ASIR, ASPR, ASMR, and ASDR, decreasing by more than 85%. Between 2010 and 2021, these indicators declined by over 90% compared to earlier periods. Joinpoint analysis showed that only the ASPR maintained a consistent downward trend throughout the study period, whereas changes in the ASIR, ASMR, and ASDR were not statistically significant. The largest reductions in ASIR and ASPR occurred between 2012 and 2015. These achievements from 2012 to 2015 can be attributed to two primary factors. First, increased political stability and enhanced support from the Global Fund to Fight AIDS, Malaria and TB and the Chinese government enabled a greater allocation of healthcare funding and facilitated the implementation of the national malaria control program (PNLP). This program included the expanded use of LLINs, IRS, IPTp, and enhanced malaria medical care (9, 30, 31). Second, mass drug administration (MDA) was introduced effectively in high-transmission areas (31, 32). MDA, which treats entire populations regardless of infection status or symptoms, aims to eliminate infection sources and requires coverage rates exceeding 80% for optimal effectiveness (31, 33). Previous research has shown that MDA significantly reduces new malaria cases and related mortality, substantially contributing to the country’s progress in malaria control (31, 32). However, after 2015, malaria incidence rose significantly until 2019, while prevalence increased markedly between 2015 and 2018. Several factors may have contributed to these trends. Climate change, particularly rising temperatures and altered rainfall patterns, has extended the malaria transmission season throughout sub-Saharan Africa (34), which poses enormous challenges to malaria control and prevention. Insecticide-resistant mosquitoes and artemisinin resistance in *Plasmodium falciparum* also pose a major threat to malaria control and elimination (35, 36). Chakir et al. reported the emergence of mosquito resistance in the Comoros, largely due to the widespread use of agricultural pesticides. They also identified potential risks arising from the introduction of artemisinin-based combination therapy (ACT)-resistant strains, partly as a result of unregulated population movement between the Comoros Islands and neighboring regions (9). Furthermore, studies found substantial genetic diversity in *Plasmodium falciparum* in the Comoros (37, 38). These genetic polymorphisms reduce parasite susceptibility to antimalarial drugs through specific mutations in the *pfcrt*, *pfmdr-1*, *pfdhfr*, and *pfdhps* genes, thereby conferring resistance to both chloroquine and artemisinin derivatives (39–41). The widespread dissemination of these drug-resistant strains can impede malaria control and elimination. Additional challenges stem from the historically narrow focus of health interventions in the Comoros, which has primarily targeted children under five and pregnant women (28, 42), inadvertently overlooking school-aged children. Cohee et al. have shown that this neglected demographic constitutes a significant parasite reservoir, potentially undermining elimination efforts and contributing to disease resurgence (25). Moreover, the previous study, conducted by Nadia et al., has identified persistent misconceptions about malaria and MDA among communities on Grande Comore Island (43). Limited public understanding of malaria’s etiology, transmission mechanisms, diagnosis, prevention, and treatment often leads to poor adherence to preventive measures and delays in seeking medical care. Misconceptions regarding MDA, especially concerns about side effects, frequently prompt medication refusal among at-risk populations. Overall, these factors create major barriers to effective malaria control and elimination in the Comoros.

The marked rise in malaria incidence from 2021 to 2022 may be attributed to two key factors. First, following wide-ranging interventions, especially MDA, which considerably reduced malaria incidence and mortality, the national malaria control center and healthcare facilities scaled back their intervention efforts. Second, a severe humanitarian crisis in Africa, precipitated by the outbreak of the global COVID-19 pandemic (4), led to disruptions in funding, technical support, and the supply of antimalarial resources in the Comoros, thereby adversely affecting malaria prevention and control efforts in the country.

Several limitations of this study should be noted. First, the analysis relied on modeled estimates from the GBD2021 database. Heterogeneity in health statistics and reporting systems across countries and regions may affect data completeness and accuracy. Varying quality in primary data sources could also influence the precision of the findings. Second, this study investigated the malaria burden only at the national level in the Comoros, without conducting subnational analyses for the three main islands (Grande Comore, Anjouan, and Mohéli). This approach may overlook potential differences in malaria burden and associated factors among the islands. Third, while the study examined overall trends and determinants of the malaria burden at a macro level, it lacked detailed data on the implementation and adherence to specific intervention measures, such as drug coverage, insecticide-treated net usage, and indoor residual spraying. In addition, other potential factors influencing malaria transmission, including socioeconomic and environmental variables, were not comprehensively quantified in this analysis.

## Conclusion

This study presents a comprehensive analysis of the malaria burden in the Comoros from 1990 to 2021. The findings highlight the need for increased attention to women under 40, school-aged children, and adults under 30. Although the Comoros has made significant progress in reducing malaria, largely due to the effective implementation of MDA alongside the PNLP, considerable improvements are still required to achieve a malaria-free status. The introduction of WHO-recommended malaria vaccines should be considered an effective preventive measure for children. Furthermore, it is crucial to establish a robust and comprehensive surveillance system that continuously monitors malaria epidemiology, disease burden indicators, vector dynamics, and antimalarial drug resistance. Disseminating knowledge about malaria prevention, treatment, and control among local communities and villages is equally vital. In addition, enhancements to the existing healthcare infrastructure are necessary to ensure that malaria control efforts remain uninterrupted even during global emergencies. Ultimately, sustained investments in malaria control are essential for the long-term success of these initiatives.

## Data Availability

Publicly available datasets were analyzed in this study. This data can be found at: https://ghdx.healthdata.org/.

## Footnotes

^1^ https://icd.who.int/browse/2024-01/mms/en

^2^ https://ghdx.healthdata.org/gbd-2021
